# A rare intramuscular osteolipoma: A case report

**DOI:** 10.1016/j.ijscr.2020.01.023

**Published:** 2020-01-25

**Authors:** Jae Hwi Han, Sung Choi, Kyung-Rak Sohn, Seong Mun Hwang

**Affiliations:** a Department of Orthopedic Surgery, Daegu Fatima Hospital, Daegu, Republic of Korea; b Department of Pathology, Daegu Fatima Hospital, Daegu, Republic of Korea

**Keywords:** Lower leg, Lipoma, Osteolipoma, Pathogenesis, Excisional biopsy, Case report

## Abstract

**Introduction:**

Lipomas are frequently presented in adults and account for almost 50% of all soft-tissue tumors. Osteolipomas are rare and usually located in the intraosseous region or adjacent to bone. It is very unusual for osteolipomas with no connection to bony structures. We report a rare intramuscular osteolipoma independent of bone tissue.

**Presentation of case:**

We report a case of a 58-year-old man with painful and progressively enlarging mass in the right lower leg. A plain X-ray and computed tomography (CT) scans revealed a large homogeneous, low-fat density mass containing an oval shape calcification without bone connection. MRI showed a circumscribed mass in the peroneus muscle with a large calcified component. The patient underwent surgical excision of the mass. Histologically, benign osteolipoma was the final diagnosis. No recurrence was observed at six months follow-up.

**Discussion:**

Lipoma is a common benign soft tissue neoplasm but osteolipoma is rare. Most cases osteolipomas are connected with bone. independent of bone tissue has been reported in very few cases. Most of them occurred in the head and neck area. The pathogenesis of osteolipoma is still not clear. Although CT and MRI are useful for differential diagnosis, care should be taken because sometimes they are indistinguishable from well-differentiated liposarcomas. Excisional biopsy is useful for definitive diagnosis.

**Conclusion:**

Although ossifying lipomas are very rare, it is important to keep them in mind when a lesion with adipose tissue in combination with ossification is encountered.

## Highlights

• Osteolipomas are rare and usually located in the intraosseous region or adjacent to bone.
• Osteolipoma with no connection to bony structures is very unusual.
• CT and MRI are useful for diagnosis but sometimes they are indistinguishable from well-differentiated liposarcomas.
• Definitive diagnosis of the lesion can easily be done with histopathologic examination and treatment is by surgical excision.

## 1 Introduction

Lipomas are frequently presented in adults and account for almost 50% of all soft-tissue tumors [1,2]. Variants of lipoma have been described according to the type of tissue present: fibrolipoma, myxolipoma, myolipoma, angiolipoma, pleomorphic lipoma, spindle-cell lipoma, angiomyolipoma [1,3,4]. In contrast lipomas with osseous or cartilaginous metaplasia are rare histological variants. Osteolipomas are less common than chondrolipomas and normally are presented in large and long term evolution lesions [3]. Osteolipomas are usually located in the intraosseous region or adjacent to bone tissue. It is very unusual for lipomas with no connection to bony structures to contain mature osseous components [1,5]. To the best of our knowledge, an intramuscular osteolipoma independent of bone tissue presented below the knee has not been previously reported in the literature. The work has been reported in line with the SCARE criteria [6].

## 2 Case presentation

A 58-year-old man presented to our hospital with a painful and progressively enlarging mass in the right lower leg. The patient noted a soft tissue mass in the anterolateral aspect of his lower leg seven years before. He had no family history, no medical history and had not any severe trauma or irradiation to the region. One month prior to presentation, his discomfort became obvious and he began experiencing pain and tenderness. The pain was dull-aching in nature and did not radiate to other regions.

On physical examination, a giant mass that was ovoid, firm, tender, well demarcated, and relatively fixed was palpated in the right peroneus muscle upper area and size was about 10 cm length and 2 cm width. The knee movements was normal. The neurological examination was within normal limits, and no lymphadenopathy was present. Laboratory data showed normal values including calcium, phosphorus, and alkaline phosphatase.

**Fig. 1:**
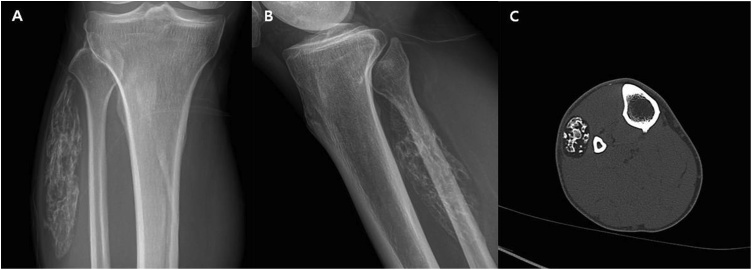
Plain X-ray (A,B) and CT (C) scan showing an ossified soft tissue mass. The density of the mass was similar to the subcutaneous fat. Diffuse ossification is seen in the center. Continuity between a tumor and fibula was not found.

**Fig. 2:**
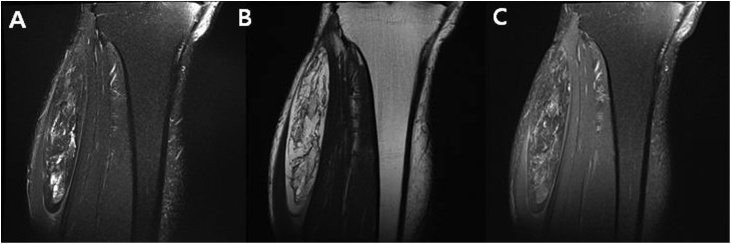
MRI showing a well defined ossified mass in the peroneous muscle. Coronal T2-weighted Fat-suppression (A), Coronal T2-weighted (B), Coronal T1-weighted Fat-suppression contrast enhanced (C).

A plain X-ray and computed tomography (CT) scans revealed a large homogeneous, low-fat density mass containing an oval shape calcification from fibula neck to fibula shaft about 11 cm length and 2 cm width. Continuity between a tumor and fibula was not found. (Fig. 1). Magnetic resonance imaging (MRI) showed a circumscribed mass in the peroneus muscle with a large calcified component (Fig. 2).

**Fig. 3:**
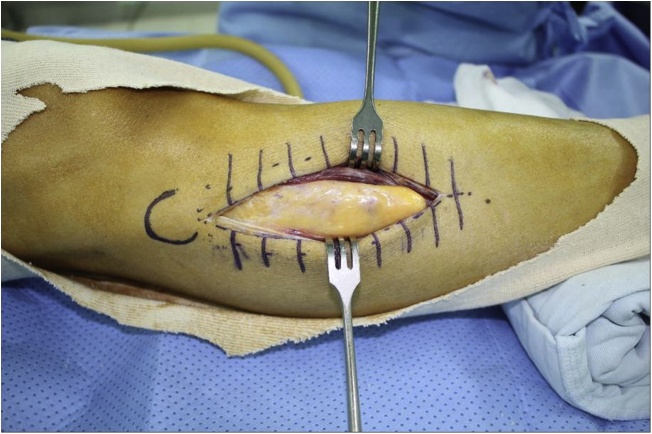
There was no continuity between the mass and the adjacent bone.

An excisional biopsy was undertaken. During the operation, a well encapsulated tumor mass was found to be located in the peroneus muscle (Fig. 3). There was no continuity between the mass and the adjacent bone. The tumor mass was removed surgically, and the incision was closed without a drain. The wound healed well, and the patient returned to daily activity after 2 days without complications.

**Fig. 4:**
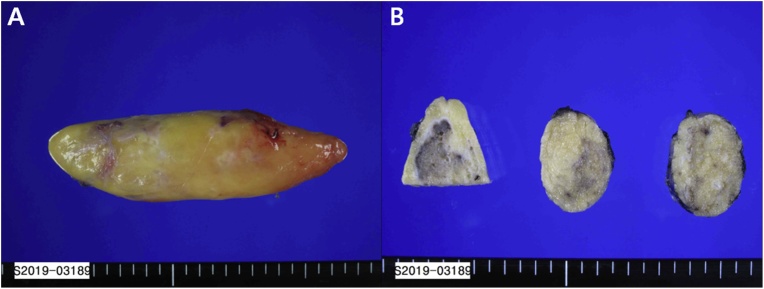
Grossly (A) mass was a well demarcated, diffusely yellowish adipose tissue mass. On sectioning (B), the cut surface consists of deep yellowish fat admixed with multiple scattered calcified white or brown bone.

Grossly, the resected specimen is a well demarcated, diffusely yellowish adipose tissue mass measuring 11 × 3.5 × 2 cm and 43 g in weight. Outer surface is diffusely smooth because of a thin fibrous capsule. On sectioning, the cut surface consists of deep yellowish fat admixed with multiple scattered calcified white or brown bone (Fig. 4).

**Fig. 5:**
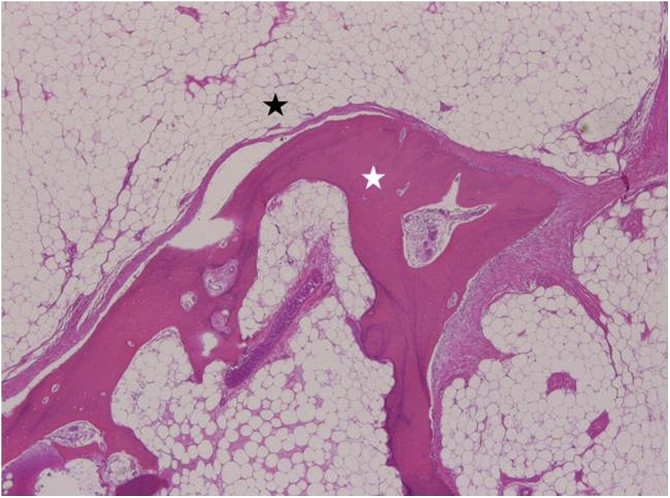
Histopathology of the mass showing mature adipose tissue (black star) and mature trabecular bone (white star).

The microscopic analyses revealed osseous trabeculae inside a mature adipose tissue. No cellular atypia or increased of mitotic figures were observed. These findings led us histopathological diagnosis of benign osteolipoma (Fig. 5).

The definitive pathological diagnosis was intramuscular osteolipoma without evidence of malignancy. No recurrence was observed at six months follow-up.

## 3 Discussion

Lipoma is a common benign soft tissue neoplasm that sometimes may have mixed tissue components. Lipomas with mixed components are named according to the type of tissue. Ossification of a lipoma was first described in 1959, and it is rarely reported [7]. In a series of 635 lipomas seen over a 5-year period, only 6 cases with ossification were found [1]. A lipoma containing mature osseous elements is called osteolipoma. The terms ossifying lipoma, osseous lipoma, and lipoma with osseous metaplasia have been used interchangeably with osteolipoma [8]. Most cases of lipomatous lesions with osseous tissue connected with bone (inside a bone or adjacent to bone) [[8], [9], [10]]. They are intraosseous lipoma or parosteal lipoma. Osteolipoma independent of bone tissue has been reported in very few cases. Most of them occurred in the head and neck area [3,[11], [12], [13], [14]]. There have been very few reports of ossifying lipomas arising either away from the head and neck or independently from bone [1,5] (Table 1). In the presented case, there was no connection between intramuscular osteolipoma and adjacent bone tissue.

**Table 1:** Reported cases of osteolipoma.

Author	Location	Connection with bone	Management	Length of follow up	Recurrence
Kumar et al. [14]	Eyelid	No	Excisional biopsy	Not described	Not described
de Castro et al. [3]	Buccal mucosa	Not described	Excisional biopsy	Not described	No
Durmaz et al. [11]	Nasopharynx	Yes	Excisional biopsy	6 months	No
Adebiyi et al. [16]	palate	Not described	Excisional biopsy	Not described	Not described
Piattelli et al. [13]	Tongue	No	Excisional biopsy	4 years	No
Kameyama et al. [12]	Neck	No	Excisional biopsy	Not described	Not described
Yang et al. [15]	Posterior Neck	No	Excisional biopsy	6 months	No
Jaiswal et al. [18]	Lumbar	Yes	Excisional biopsy	3 weeks	Not described
Yabe et al. [10]	SC joint	No	Excisional biopsy	Not described	No
Obermann et al. [8]	Scapula	Yes	Excisional biopsy	Not described	Not described
Demiralp et al. [5]	Inguinal	No	Excisional biopsy	18 months	No
Electricwala et al. [19]	Femur	Yes	Excisional biopsy	8 months	No
Heffernan et al. [1]	Thigh	No	Wide excisional biopsy	Not described	Not described

The pathogenesis of osteolipoma is still not clear. Two main theories exist for the pathogenesis of osteolipomas [3,5]. First, These tumors appear to be of mesenchymal origin, which is derived from pluripotent cells then it may be called as benign mesenchymoma [3]. This pathology is defined as a rare soft tissue lesion composed of fibrous tissue associated with two or more types of mesenchymal cells well differentiated, that would not normally be found in the same area [3]. According to the second theory, ossification may also have been induced by poor nutritional supply in the centre of a large lipoma after repetitive trauma, metabolic changes, or ischemia leading to transformation of fibroblasts into osteoblasts [5,15]. Our histological finding does not clarify which of the two hypotheses is true.

Congenital malformations of bones, benign tumors containing bony tissue (teratomas, dermoid), secondary ossification due to trauma, liposarcoma with metaplastic changes should be considered in the differential diagnosis. The use of CT scanning provides excellent visualization of the calcified or ossified components of a lipoma and confirmation of proximity to adjacent bone, and MR imaging can also provide detailed information that is useful for further evaluation [16,17]. However, on Magnetic Resonance Imaging, lipoma variants have unusual features on imaging studies. Intralesional non-adipose components can confound the correct imaging diagnosis because they can mimic findings associated with well-differentiated liposarcomas [17]. Definitive diagnosis of the lesion can easily be done with histopathologic examination and treatment is by surgical excision [18]. Lipomas with osseous changes have the same prognosis as lipomas [8].

## 4 Conclusion

Although ossifying lipomas are very rare, it is important to keep them in mind when a lesion with adipose tissue in combination with ossification is encountered.

## Sources of funding

No funding was requested.

## Ethical approval

This is a case report study, no ethical approval was needed. On the other hand, the patient had been informed and gave their consent regarding this publication.

## Consent

Written informed consent was obtained from the patient for publication of this case report.

## Author contribution

JH Han performed the operation and perioperative management of the patient. JH Han also acquired and interpreted the data and drafted the manuscript. SM Hwang participated in the operation, perioperative management of the patient. S Choi revision of the manuscript. KR Sohn reviewed pathological findings.

## Registration of research studies

This manuscript is not a human study, but a case report.

## Guarantor

Jae Hwi Han.

## Provenance and peer review

Not commissioned, externally peer-reviewed.

## Declaration of Competing Interest

No conflict of interest for all authors.
